# Siderophores as “Trojan Horses”: tackling multidrug resistance?

**DOI:** 10.3389/fmicb.2014.00290

**Published:** 2014-06-12

**Authors:** Carla C. C. R. de Carvalho, Pedro Fernandes

**Affiliations:** ^1^Department of Bioengineering, Centre for Biological and Chemical Engineering, Institute of Biotechnology and Bioengineering, Instituto Superior Técnico, Universidade de LisboaLisboa, Portugal; ^2^Faculdade de Engenharia, Universidade Lusófona de Humanidades e TecnologiasLisboa, Portugal

**Keywords:** iron transport, siderophore conjugates, anti-bacterial acitivity, multidrug resistance, drug delivery

Microbial drug resistance is partly due to hindered diffusion through the membrane of microbial cells and active transport mechanisms. An approach to counter such resistance uses the bacterial iron transport system. Extracellular free iron is scarce in vertebrates, yet essential for microbial growth (Anderson et al., 2012). A mechanism displayed by microbial pathogens to cope with iron scarcity involves the production of siderophores (Skaar, 2010). These low molecular weight molecules bear an affinity to iron that exceeds by several orders of magnitude that of transferrin, the main protein in blood for iron transport (Clifton et al., 2009). Under iron starvation, siderophores are excreted, scavenge ferric ions and the complex is shuttled inside the cell. The pathway differs for gram-negative and gram-positive strains, in a mechanism better known for the former (Fukushima et al., 2013). The Trojan horse approach (THA) relies on the iron-siderophore uptake system to deliver an antibiotic payload (Figure 1), a mechanism displayed by several bacteria, through the production of e.g., albomycins, ferrymicins, and salmycins. These sideromycins consist of naturally occurring hydroxamate type of siderophores, covalently linked to an antibiotic moiety (Möllmann et al., 2009). Aiming to improve antibiotic uptake by pathogenic bacteria, efforts have been made in the design of siderophore-antibiotic conjugates (Page, 2013). Typically this involves a catechol/hydroxamate siderophore analog and a β-lactam drug. Care is required so that: the mechanism of siderophore recognition and uptake is not hampered; a suitable linker is used, thus the conjugate is stable in extracellular environment but the drug is released intracellularly by enzyme action, in either the cytoplasm or the periplasm, the latter often required to maximize the activity of the conjugate (Braun et al., 1983). Interesting developments have occurred in the design of siderophore-drug (SD) conjugates (Page, 2013; Mislin and Schalk, 2014), up to the point where a siderophore monosulfactam, BAL30072, gave promising results enough for clinical trials to be performed, being currently at phase 1 (Butler et al., 2013). This type of compounds conjugates a lactam, or similar, with a siderophore-mimicking small molecule. BAL30072 combines a dihydroxypyridone moiety, the oxyiminoacyl side chain enabling easy access to the bacterial cell through the iron uptake system, and a monocyclic β-lactam antibiotic moiety. The latter has reduced susceptibility to inactivation promoted by different β-lactamases (Hofer et al., 2013). BAL30072 retained activity in the presence of strains producing class C carbepenemases, unlike third-generation cephalosporins and aztreonam and displayed antimicrobial activity against a significant array of Gram negative strains, among them (multi)drug resistant *Burkholderia pseudomallei*, *P. aeruginosa*, and *Acinetobacter baumannii* (Mushtaq et al., 2010; Page et al., 2010; Mima et al., 2011; Higgins et al., 2012). The conjugate proved effective toward 80% of the *A. baumanii* strains tested using an *in-vivo* rat soft-tissue infection model (Russo et al., 2011). *In-vitro* combinations of BAL30072 and carbapenems proved more effective than individual agents against multidrug resistant (MDR) Gram-negative strains. Additive and synergistic effects on anti-microbial activity were observed, particularly in *Enterobacteriaceae* and *P. aeruginosa*. The latter was ascribed to the affinity of BAL30072 and carbapenems for the target of β-lactam drugs: the membrane-bound penicillin-binding proteins (PBPs) in the strains tested. The synergistic effect observed *in-vitro* was translated with efficacy *in-vivo* using animal models of septicaemia, where the challenging strains included *A. baumannii*, *P. aeruginosa*, and *S. marcescens* (Hofer et al., 2013). *In-vitro* anti-bacterial activity against the pathogen *A. baumannii ATCC 17961* was also reported recently for a biscatecholate-monohydroxamate sideromycin linked by a succinyl residue to a carbacephalosporin antibiotic. The conjugate allowed for a MIC of 0.125 μM, compared to 0.25 μM and over 128 μM for ciprofloxacin and Loracarbef, respectively (Wencewicz and Miller, 2013). The parent siderophores were antagonists for the conjugate and its antibacterial activity inversely related to the concentration of Fe(III) in the media.

**Figure 1:**
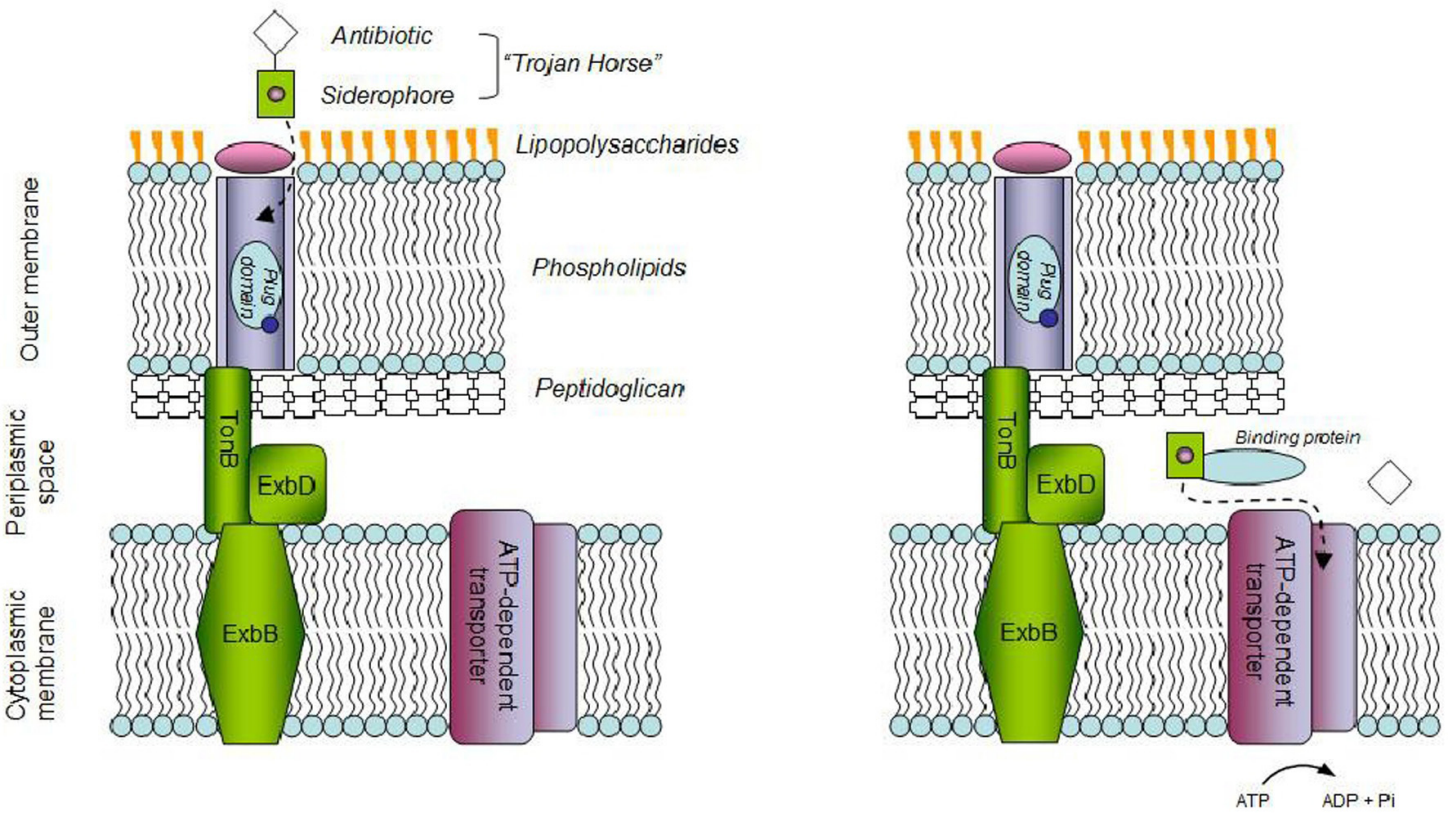
**Schematic of the “Trojan horse” strategy in Gram-negative bacteria**. Antibiotic bound-siderophores bind to outer-memberane receptors which help their transport to the periplasmic space upon interaction with the Ton complex. In the periplasm, siderophores are sequestered by periplasmic-binding proteins which deliver them to ATP-dependent transporters which help their entrance into the cell. Antibiotics which would not pass cellular membranes otherwise, can therefore be released.

Most of the research within the THA for drug delivery has relied on β-lactams. Yet, the use of lactivicin and derivatives coupled to a phthalimide group, recognizable by a bacterial siderophore receptor, and hence transported into the cell, was recently established as a promising inhibitor of PBPs. The phthalimide-lactivicin-based conjugate may use a wider set of Ton-B receptors than those related to hydroxypyridone-β-lactams (Starr et al., 2014).

Application of SD conjugates for tackling MDR Gram-positive strains has also been considered. In a recent work, a synthetic trihydroxamate-ciprofloxacin (a fluoroquinolone) conjugate displayed antibacterial activity against *Staphylococcus aureus* SG511 (MIC of 1 μM, twice that of the parent antibiotic and similar to Loracarbef). The authors established that hydroxamate-fluoroquinolone conjugates used active transport to deliver the payload to their cytoplasmic DNA gyrase target, and that the full trihydroxamate backbone was required for such active transport, which oppositely denied β-lactam syderomycin access to PBPs (Wencewicz et al., 2013).

*Mycobacterium tuberculosis* (Mtb) presents another challenge for the THA. Three synthesized acetylated mycobactin T molecules displayed selective inhibition ability against Mtb H37Rv (MIC_90_ within 0.02–0.88 μM in 7H12 medium), but showed no inhibitory action over a wide range of Gram-positive and Gram-negative strains. This behavior was ascribed to the selective nature of iron transport, and the analogs were considered promising platforms for further developments in conjugate assembly (Juárez-Hernández et al., 2012). Within this methodology, a mycobactin T analog was synthesized to enable linking to an artemisinin payload, which has anti-malarial activity, but no anti-tuberculosis activity. The conjugate displayed high anti-tuberculosis activity against MDR Mtb but no activity against several fast-growing mycobacteria. The toxicity of the conjugate was ascribed to its ability to fuel the formation of hydroxyl radicals in Mtb, in a mechanism that differs from other THA SD (Miller et al., 2011). The THA based SD conjugate against pathogenic bacteria is evolving but is far from its full potential. Increasing know-how at molecular level of the mechanisms of transport through the cell wall, the structure of siderophore receptors and of the targets for the payload, the design of synthetic siderophore analogs and particularly of the linker, which involves also detailed insight into enzyme/substrate interaction, is critical. Finally, *in-vivo* tests are required to validate the most promising results *in-vitro*.

## Conflict of interest statement

The authors declare that the research was conducted in the absence of any commercial or financial relationships that could be construed as a potential conflict of interest.
